# A Randomized Trial Assessing the Effectiveness of Ezetimibe in South Asian Canadians with Coronary Artery Disease or Diabetes: The INFINITY Study

**DOI:** 10.1155/2012/103728

**Published:** 2012-12-06

**Authors:** Mina Madan, Tasnim Vira, Emmanouil Rampakakis, Anup Gupta, Anil Khithani, Lyn Balleza, Julie Vaillancourt, Stella Boukas, John Sampalis, Emidio de Carolis

**Affiliations:** ^1^Schulich Heart Centre, Sunnybrook Health Sciences Centre, D380-2075 Bayview, Avenue, Toronto, ON, Canada M4N 3M5; ^2^JSS Medical Research Inc., 9400 Henri-Bourassa W., St-Laurent, PQ, Canada H4S 1N8; ^3^McGill University, Montreal, PQ, Canada; ^4^Division of Cardiology, Toronto East General Hospital, 751 Pape Avenue, Toronto, ON, Toronto East General Hospital, Canada M4K 3T1; ^5^3430 Finch Avenue East, Scarborough, ON, Canada M1W 2R5; ^6^Merck Canada Inc., 16711 Trans-Canada Hwy., Kirkland, PQ, Canada H9H 3L1

## Abstract

*Background*. There is a paucity of data regarding the effectiveness and safety of lipid-lowering treatments among South-Asian patients. *Methods*. Sixty-four South-Asian Canadians with coronary artery disease or diabetes and persistent hypercholesterolemia on statin therapy, were randomized to ezetimibe 10 mg/day co-administered with statin therapy (EZE + Statin) or doubling their current statin dose (STAT^2^). Primary outcome was the proportion of patients achieving target LDL-C (<2.0 mmol/L) after 6 weeks. Secondary outcomes included the change in lipid profile and the incidence of treatment-emergent adverse events through 12 weeks. Exploratory markers for vascular inflammation were assessed at baseline and 12 weeks. *Results*. At 6 weeks, the primary outcome was significantly higher among the EZE + Statin patients (68% versus 36%; *P* = 0.031) with an OR (95% CI) of 3.97 (1.19, 13.18) upon accounting for baseline LDL-C and adjusting for age. At 12 weeks, 76% of EZE + Statin patients achieved target LDL-C compared to 48% (*P* = 0.047) of the STAT^2^ patients (adjusted OR (95% CI) = 3.31 (1.01,10.89)). No significant between-group differences in exploratory markers were observed with the exception of CRP. *Conclusions*. Patients receiving ezetimibe and statin were more likely to achieve target LDL-C after 6 and 12 weeks compared to patients doubling their statin dose. Ezetimibe/statin combination therapy was well tolerated among this cohort of South-Asian Canadians, without safety concerns.

## 1. Introduction

 Despite a steady decline in coronary artery disease (CAD)-related mortality over the past 25 years, CAD remains a leading cause of death among Canadians [1]. The direct association between higher serum concentrations of low-density lipoprotein cholesterol (LDL-C) and increased risk of CAD is well known [2–8]. In patients with primary hypercholesterolemia, the main treatment goal is lowering LDL-C to reduce CAD risk [9]. The 2009 Canadian dyslipidemia guidelines recommend treatment of patients with CAD or CAD risk equivalent conditions with an LDL-C level ≥ 2.0 mmol/L with lipid-lowering agents [9]. With declining LDL treatment targets, multiple therapies may be required to achieve these goals.

Hydroxymethylglutaryl-coenzyme-A (HMG-CoA) redu-ctase inhibitors (statins) are considered first-line therapy for hypercholesterolemia [9]. For patients not achieving their target LDL-C levels, increasing the statin dose, switching to another statin, or coadministration of complementary drugs to statins may be tried. The cholesterol absorption inhibitor, ezetimibe, reduces cholesterol absorption in the small intestine [10]. It may be used alone, when statins are not tolerated, or together with statins for synergy [10]. Furthermore, ezetimibe may be statin-sparing, if high-dose statin therapy resulted in adverse events such as myopathy or impaired liver function. Due to their complementary mechanisms of action, the coadministration of ezetimibe with statin should deliver high LDL-C lowering efficacy at relatively lower doses of statin.

South Asians are a prominent ethnic group in Canada (Canadians with ethnic background from India, Pakistan, Nepal, Bangladesh, or Sri Lanka) with a high incidence of metabolic syndrome and CAD [11, 12]. Genetic predisposition, dietary habits, or assuming a western diet may affect the incidence of hypercholesterolemia among South Asians and their response to treatment [11, 12]. Data assessing and comparing lipid-lowering treatments in this ethnic group are limited. The current study compared the effectiveness and safety of ezetimibe 10 mg daily coadministered with current statin dosing versus a strategy of doubling the current statin dose in South Asian Canadians with either established CAD or diabetes (high-risk patients), who remained above target LDL-C while taking statin therapy.

## 2. Methods

This was a 12-week multicenter, prospective, randomized, and open-label clinical trial. Patients were included if they: (1) were Canadians with ethnic background from India, Pakistan, Nepal, Bangladesh, or Sri Lanka; (2) had primary hypercholesterolemia and known CAD or diabetes, either by history, angiographic, or laboratory evidence; (3) had a LDL-C ≥ 2.0 mmol/L while on any statin below maximum daily dose for a minimum of 4 weeks before enrollment. Statin-naïve patients and patients unable to have their statin dose doubled due to maximal statin dosing already, or tolerability/safety concerns, were excluded. Additional exclusion criteria were: (i) treatment with fish oils, cholestin, bile acid sequestrants, niacin, or fibrates, and; (ii) active liver disease, uncontrolled endocrine illness, kidney disease, or creatine kinase (CK) > 50% above the upper limit of normal (ULN). The study was approved by an independent central research ethics board (Canadian SHIELD Ethics Review Board).

### 2.1. Visits

**Figure 1:**
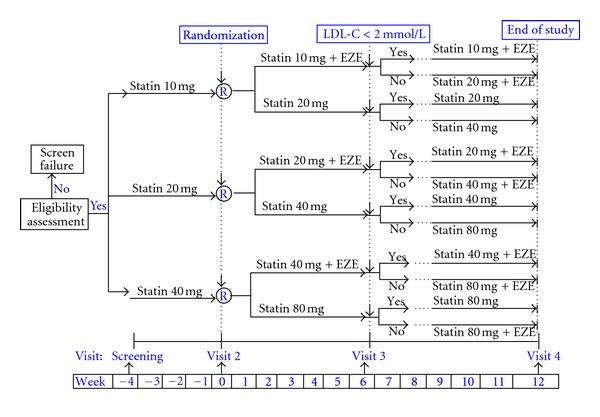
Study design.

After providing informed consent, study participants attended 4 clinic visits. At Visit 1 the fasting lipid profile, CK, and liver function parameters were assessed. Eligible patients entered a 4-week stabilization phase during which they continued taking their current statin dose (Figure 1). At Visit 2, eligibility for randomization was confirmed with another fasting lipid profile. Patients who remained eligible (LDL-C ≥ 2.0 mmol/L) were randomized (1 : 1 ratio) to receive either ezetimibe 10 mg daily co-administered with current statin dosing (EZE + Statin) or doubling of current statin dose (STAT²). Randomization was stratified according to baseline LDL-C level (≥2.0 mmol/L and <3.0 mmol/L, ≥3.0 mmol/L and <3.5 mmol/L, and ≥3.5 mmol/L) and centrally coordinated using an interactive voice response system. Bloodwork for exploratory markers (C-reactive protein (CRP), interleukin-6 (IL-6), apolipoprotein B, apolipoprotein A1, adiponectin, and leptin) was obtained at Visit 2. After 6 weeks (Visit 3), a brief exam, blood draw for fasting lipid profile, and review of any adverse events occurred. For EZE + Statin patients, if LDL-C levels were 2.0 mmol/L, the statin dose was doubled for the next 6 weeks. For STAT^2^ patients with LDL-C 2.0 mmol/L, the statin dose was again doubled for the next 6 weeks. If the patient was already at maximum statin dose, ezetimibe 10 mg/day could be added at the physician's discretion (crossover). At week 12 (Visit 4), patients underwent a brief exam, review of adverse events, fasting lipid profile, and repeat exploratory markers. STAT^2^ patients, who crossed over to ezetimibe therapy, underwent exploratory marker testing at Visit 3, before ezetimibe was started.

### 2.2. Definitions

The primary endpoint was the proportion of patients in each treatment group achieving target LDL-C concentration (<2.0 mmol/L) after 6 weeks of therapy. Main secondary endpoints included the proportion of patients achieving target LDL-C concentration (<2.0 mmol/L) at 12 weeks, the absolute and percent change in lipid parameters during the study, the incidence of treatment-emergent adverse events, and exploratory markers levels at baseline and 12 weeks. 

### 2.3. Sample Size Calculation

Sample size calculation was based on the primary outcome measure, namely, the achievement of target LDL-C at 6 weeks. In the EZE (STAT)^2^ trial [13], 40% of the patients treated with current statin co-administered with ezetimibe achieved LDL-C < 2.0 mmol/L at 6 weeks compared with 18% of those treated with doubling of the current statin dose. In order to detect a similar magnitude of difference for this outcome, with 5% significance and 90% power, a total of 111 patients per group were required. Since recruitment and retention difficulties for ethnic minorities have been reported in the literature, a 25% attrition rate was initially assumed, resulting in a target sample size of 150 patients per group for a total of 300 patients. Due to challenges related to recruitment and enrollment, the target sample size was not achieved. Post hoc power calculation of the effect size of the primary endpoint for 64 patients (34 in EZE + Statin group and 30 in STAT^2^ group) with a 95% level of precision (2-tailed alpha of 0.05) resulted in 74% power. However, due to the small sample size the results of this study must be interpreted with caution.

### 2.4. Statistical Analysis

All analyses were performed in the intent-to-treat (ITT) population including all patients who were randomized. There was no imputation of missing values with the exception of the dropout equals failure approach for the target LDL-C achievement. Frequencies and proportions were reported for categorical variables; means and standard deviation (and median, minimum, maximum values for nonnormally distributed parameters) were reported for continuous variables. Between-group comparisons were assessed for statistical significance with the Chi-square test for categorical variables, the independent-samples Student's *t*-test for continuous variables and the Mann-Whitney *U* nonparametric test for continuous variables not normally distributed. Logistic regression was used to assess between-group differences in LDL-C target achievement while accounting for baseline LDL-C values (≥2.0 mmol/L to <3.0 mmol/L versus ≥3.0 mmol/L to <3.5 mmol/L versus ≥3.5 mmol/L as per stratified randomization) and adjusting for potential confounders. Within-group comparisons were performed using the one-sample *t*-test. All statistical tests were two-sided with an alpha level of 0.05. All analyses were performed using SPSS version 12.0 (SPSS, Chicago, IL, USA).

## 3. Results

### 3.1. Study Participants

**Figure 2:**
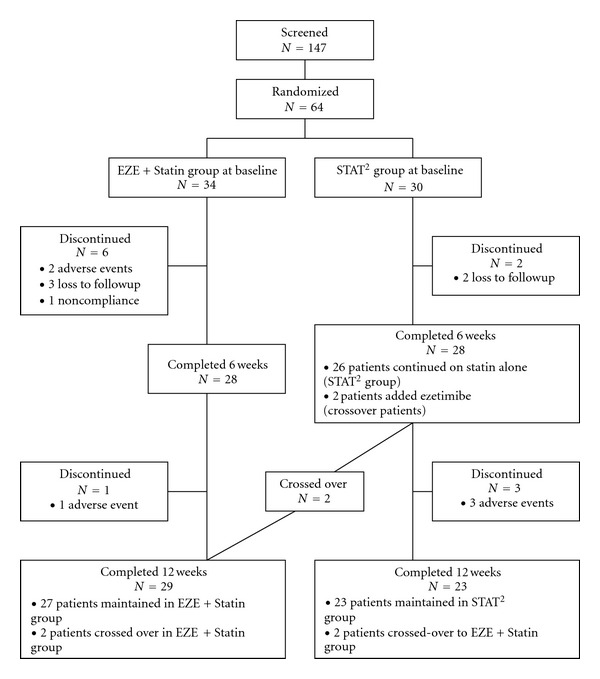
Enrollment, randomization, and followup of study participants.

From 147 patients screened, 64 (44%) met the inclusion/exclusion criteria and were randomized (Figure 2). The most common reason for exclusion was a Baseline LDL-C <2.0 mmol/L. Among the 64 patients enrolled, 56 (88%) patients completed the 6-week assessment and 52 (81%) completed the 12-week assessment. During the course of the study, there were 12 patients who were prematurely discontinued; 5 were lost to followup, 6 had adverse events, and 1 patient was noncompliant. Of the 28 STAT^2^ patients who completed 6 weeks of treatment, 2 were eligible for a crossover since they had not achieved LDL-C target while treated with the maximum statin dose (80 mg) for 6 weeks. Both of them had ezetimibe added to their statin treatment and were, therefore, crossed over to the EZE + Statin group between 6 and 12 weeks. The final patient disposition after accounting for the 2 crossover patients was 29 and 23 for the EZE + Statin and STAT^2^ groups, respectively. 

### 3.2. Baseline Characteristics

Roughly half the participants had known CAD, and 2/3 of the study cohort was diabetic (Table 1). Most patients originated from India or Sri Lanka. About 1/3 of the overall cohort had an elevated BMI, or metabolic syndrome; abdominal obesity was common. Patients randomized to EZE + Statin were older than STAT^2^ patients (60 versus 54 years, *P* = 0.048). Although not specifically mandated by protocol, all patients were taking atorvastatin (10 mg, 20 mg, or 40 mg) at baseline.

## 4. Lipid Profile

Tables 2 and 3 describe the lipid profile by patient group throughout the study. Overall, lipid parameters were comparable between groups at baseline (Table 2). Baseline LDL-C levels were slightly lower, but not clinically different, in the EZE + Statin group compared to the STAT^2^ group. Although not statistically different, greater improvements from baseline in LDL-C, total cholesterol, triglycerides, and TC/HDL-C ratio were observed in the EZE + Statin group compared to the STAT^2^ group upon 6 and 12 weeks of treatment (Table 3). Sensitivity analysis excluding the two crossed over patients from the 12-week time point had no significant impact on the results. 

**Figure 3:**
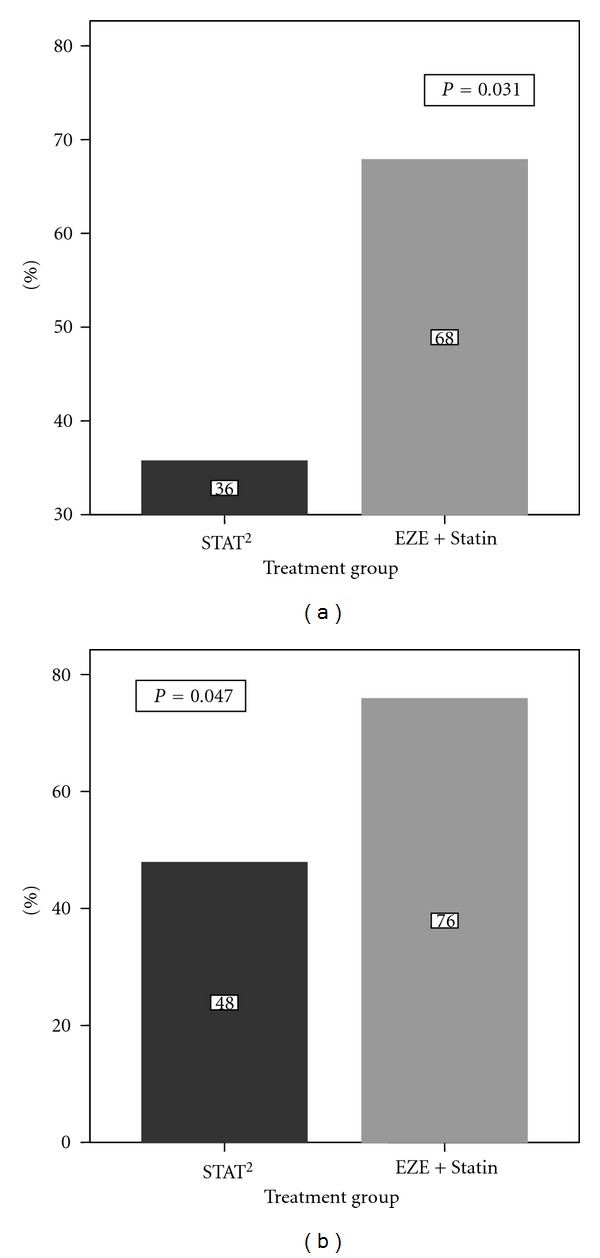
(a) Primary endpoint: percentage of patients achieving target LDL-C (<2.0 mmol/L) at 6 weeks (b) Percentage of patients achieving target LDL-C (<2.0 mmol/L) at 12 weeks.

Among patients completing 6 weeks of treatment, target LDL-C (<2.0 mmol/L) achievement was significantly higher among EZE + Statin patients compared to the STAT^2^ group (68 versus 36%, *P* = 0.031) (Figure 3(a)) with an odds ratio of 3.97 (95% CI: 1.19–13.18) upon accounting for baseline stratified LDL-C levels and adjusting for age. At 12 weeks, 76% of EZE + Statin patients had achieved target LDL-C levels compared to 48% of STAT^2^ patients (*P* = 0.047, Figure 3(b)). Inclusion of baseline stratified LDL-C levels and age as covariates gave an OR (95%CI) of 3.58 (1.02, 12.54).

In a more conservative approach where patients who had dropped out were accounted as treatment failure (not achieving LDL-C level <2.0 mmol/L), 56% versus 33% of patients in the EZE + Statin versus STAT^2^ group (adjusted OR (95%CI) = 2.39 (0.80, 7.16)) and 61% versus 39% (adjusted OR (95% CI) = 2.59 (0.88, 6.64)) achieved target LDL-C at 6 weeks and 12 weeks, respectively. 

After 6 weeks of treatment, 18 STAT^2^ patients required a statin dose increase, of whom 2 crossed over to EZE + Statin combination therapy, compared to 9 patients in the EZE + Statin group (64% versus 32%, *P* = 0.031). By 12 weeks, 44% of uptitrated patients in both arms achieved target LDL-C levels. 

### 4.1. Safety

A total of 21 nonserious adverse events were reported by 14 (21.9) patients, 4 of 34 (11.8%) patients in the EZE + Statin group and 10 of 30 (33.3%) patients in the STAT^2^ group. The most common findings were abdominal discomfort, CK elevation, and myalgias (Table 4). No patient had an increase in alanine aminotransferase or CK >3 XULN during the study. There were two serious adverse events reported; one unstable angina event, occurring in a STAT^2^ patient, and one cerebrovascular accident resulting in death before randomization.

### 4.2. Exploratory Markers

Baseline median CRP levels were normal in both study groups (with >3.0 mg/L indicating an increased risk of future cardiovascular events [14]; Table 5). Median CRP levels were significantly lower at 12 weeks among patients in the EZE + Statin group compared to the STAT^2^ group. Baseline median IL-6 levels were normal (with >3.1 pg/mL indicating an increased risk of future cardiovascular events [15]). Numerically, IL-6 became further elevated over 12 weeks among patients in the STAT^2^ patients, and to a lesser extent in the EZE + Statin group. Apolipoprotein B levels were elevated at baseline in both groups (optimal levels <0.80 g/L among high-risk patients [9]). We observed a decrease in Apolipoprotein B in both treatment arms over 12 weeks. apolipoprotein A1 was relatively unchanged by either treatment strategy. The baseline ApoB/ApoA1 ratio was already optimal (optimal ratio < 0.8 [9]) and further decreased, albeit nonsignificantly in both groups. Adiponectin levels rose slightly in EZE + Statin group and fell in STAT^2^ group. Leptin levels rose in both treatment groups, but more so in the STAT^2^ group. Other than CRP level, there were no significant differences between the two groups for all exploratory parameters at baseline and 12 weeks. 

## 5. Discussion

South Asians are among the fastest growing immigrant populations in Canada. By 2017, they may represent the largest ethnic minority in the country, reaching 1.7 million [12, 16]. South Asians have a very high prevalence of CAD, particularly at younger ages, in the absence of traditional risk factors, compared with the general population. The higher CAD risk in this population may be related in part to a higher prevalence of insulin resistance, the metabolic syndrome, and diabetes. In the case of type 2 diabetes, they are 3 times more likely to develop this condition compared to Caucasians, and the disease manifests at a younger age relative to the general population [12, 17]. Aggressive primary and secondary prevention strategies are warranted in South Asians to address the high rates of morbidity and mortality associated with CAD and diabetes in this ethnic group. 

While the elevated risk for cardiovascular events and diabetes among South Asians has been well documented, few studies have examined actual strategies to mitigate risk in this ethnic group. In this randomized trial involving South Asian Canadians with either established CAD or diabetes (high risk patients), we demonstrated that a strategy of using ezetimibe 10 mg daily co-administered with statin therapy was more effective in achieving a target LDL-C < 2.0 mmol/L at 6 and 12 weeks than a strategy of doubling the statin therapy. Although not statistically significant, we observed that percentage changes in total cholesterol, LDL-C, triglycerides, and total cholesterol/HDL-C were numerically higher in the EZE + Statin group. Ezetimibe therapy was well tolerated in this study, with a low incidence of adverse events. To our knowledge, no prior studies have evaluated an ezetimibe-based strategy among South Asians. 

The first lipid-lowering study performed exclusively among South Asians was the Investigation of Rosuvastatin in South Asians (IRIS) study [18]. IRIS was a 6-week randomized open-label trial comparing rosuvastatin and atorvastatin in 740 South Asians with hypercholesterolemia (66% of the patients were at high risk for CHD with a National Cholesterol Education Program Adult Treatment Panel III treatment goal of <2.6 mmol/L and 40% had diabetes). LDL-C decreased by 45% with rosuvastatin 10 mg versus 40% with atorvastatin 10 mg (*P* = 0.0023) and by 50% with rosuvastatin 20 mg versus 47% with atorvastatin 20 mg (*P* value not significant).

Among high-risk patients at 6 weeks, 42% and 56% reached an LDL-C goal of <1.8 mmol/L with rosuvastatin 10 and 20 mg compared with 18% and 42% with atorvastatin 10 and 20 mg, respectively. A significantly greater differential response of rosuvastatin 10 or 20 mg compared with atorvastatin 20 mg was not observed. Similar results were noted in statin trials among African American and Hispanic populations, respectively [19, 20]. The incidence of any adverse event was roughly 30% overall, the most common adverse events were myalgias, constipation, headache, and arthralgia (all <3%). Discontinuation due to adverse events was rare (2.4% rosuvastatin and 0.8% atorvastatin). No patient had hepatic dysfunction, and only 1 patient taking atorvastatin had markedly elevated CK levels (>10XULN). 

Statins may exhibit altered metabolism and efficacy in some Asian populations (predominantly East Asian) [21]. The potential mechanisms of heightened response to statins in East Asians are related to genetically-based differences in the metabolism of statins at the level of hepatic enzymes and drug transporters. Studies indicate that lower statin doses achieve lipid improvements in East Asian patients comparable with those observed with higher doses in Caucasians [21]. The 2006 Canadian dyslipidemia guidelines suggested using lower statin doses in all Asians [22]. The Prospective Assessment of Cardiovascular Risk and Treatment of Canadians of Varying Ethnicity (PRACTICE) registry evaluated effects of statins in South Asian and Caucasian patients with established CAD [23]. After 6 weeks of treatment, atorvastatin 20 mg/d produced similar decreases in LDL-C in South Asian (43%) and Caucasian (41%) patients and raised HDL-C by 19% in South Asians and by 12% in Caucasians. Six weeks of treatment with simvastatin 20 mg/d reduced LDL-C by 35% in South Asians and by 37% in Caucasian patients while raising HDL-C by 12% in both groups. These results indicate that both atorvastatin and simvastatin modulate LDL-C and HDL-C levels to a similar degree in both South Asians and Caucasians. Accordingly, the 2009 Canadian dyslipidemia guidelines no longer suggest differential dosing for South Asian patients [9]. 

While statins are considered a mainstay in the treatment of dyslipidemia, there remain high-risk patients who are unable to achieve target LDL-C with high-dose statins alone, or those patients in whom statin therapy results in significant side effects. Our study adds to the body of literature finding the ezetimibe + statin approach to be an effective and well-tolerated strategy [10, 24]. Although not specifically tested in our study, this strategy may be very helpful in achieving the LDL-C target among South Asians who are unable to tolerate high-dose statin therapy (statin-sparing). 

### 5.1. Exploratory Markers

The role of inflammation in the development, and progression of CAD is increasingly recognized [14, 15, 25–27]. We measured 6 vascular markers potentially associated with CAD. Apolipoprotein B was elevated in our study cohort, confirming the higher risk for future cardiovascular events among these patients. Apolipoprotein B declined during the study, in both treatment arms, as might be expected among patients receiving cholesterol-lowering therapy. The 2009 Canadian dyslipidemia guidelines recommend Apolipoprotein B as the primary alternate target to LDL-C [9]. Many experts consider apolipoprotein B a better index of the adequacy of LDL-lowering therapy than LDL-C. The widespread measurement of Apolipoprotein B remains fairly limited by clinicians and most laboratories [9]. Baseline levels of CRP were normal in this study, but declined significantly among those patients in the EZE + Statin group. Whether this could be interpreted as a differential anti-inflammatory potential for the ezetimibe + statin strategy would require further study. The ApoB/ApoA1 ratio also declined over 12 weeks, as might be expected among patients receiving cholesterol-lowering therapy. Low levels of adiponectin and high levels of leptin are associated with increased cardiovascular risk [11]. Although not statistically different, more decline in adiponectin and greater rise in leptin was observed among the STAT^2^ patients. Our low sample size makes it difficult to draw definitive conclusions from these data. 

### 5.2. Limitations

Our results must be interpreted with caution due to the small sample size recruited. Although we observed significant improvements in several important lipid parameters, the lack of significant change in several exploratory markers during followup may be because the study was underpowered to detect these differences. Furthermore, the evaluation of stable outpatients at risk likely limited our ability to identify large differences in these markers over time. The optimal baseline ApoB/ApoA1 ratio may be an indicator of this. The open-label study design may have biased the assessment or reporting of adverse events; however, the incidence of adverse events was comparable to other studies involving predominantly Caucasians. Because all subjects were taking atorvastatin, we cannot comment on the efficacy of other statins combined with ezetimibe. 

In conducting research dedicated to one specific ethnic group, we encountered several challenges related to recruitment, enrollment, followup, and retention. Specifically, we faced challenges with willingness to participate, difficulty comprehending the concept of a randomized trial, noncompliance, and withdrawal due to lack of transportation to clinic visits because of dependence on younger working family members. Our experience lead to inability to recruit the intended sample size (64 patients enrolled versus 300 initially planned) and a dropout rate of 19% highlighting some of the difficulties in obtaining meaningful participation of ethnic minorities in clinical studies and reducing the external validity of the findings. These difficulties for ethnic minorities have been previously reported in the literature [28–31]. The role of novel methods for recruitment and followup (such as visiting community centers and conducting home visits) should be explored for such patients to improve their inclusion in important studies. 

## 6. Conclusions

South Asian Canadians treated with co-administration of ezetimibe and a statin were more likely to achieve target LDL-cholesterol levels after 6 and 12 weeks of treatment compared to patients doubling statin monotherapy. The safety profile of ezetimibe/statin combination therapy was similar to that reported in the literature for other patient populations.

## Figures and Tables

**Table 1:** Baseline Characteristics.

Characteristic	EZE + Statin	STAT^2^	*P* Value
	*n* = 34	*n* = 30	(Between-Group)
Age, years, mean ± SD	60 ± 10	54 ± 13	0.048
Women, *n* (%)	14 (41.2)	9 (30.0)	0.437
Weight, kg, mean ± SD	71.5 ± 12.7	74.8 ± 13.4	0.310
Overweight (Body Mass Index > 27 kg/m^2^), *n* (%)	9 (26.5)	11 (36.7)	0.380
Country of Ethnic Origin, *n* (%)			0.660
India	14 (41.2)	10 (33.3)	
Pakistan	5 (14.7)	6 (20.0)	
Sri Lanka	13 (38.2)	10 (33.3)	
Other	2 (5.9)	4 (13.3)	
Known Coronary Artery Disease, *n* (%)	16 (47.1)	15 (50.0)	0.814
Diabetes*, *n* (%)	22 (64.7)	21 (70.0)	0.653
Hypertension*, *n* (%)	20 (58.8)	13 (43.3)	0.216
Current smoker, *n* (%)	1 (2.9)	3 (10.0)	0.506
Metabolic Syndrome**, *n* (%)	9 (26.5)	8 (26.7)	>0.999
Abdominal obesity	21 (61.2)	21 (70.0)	
Elevated triglycerides	9 (26.5)	6 (20.0)	
Elevated fasting blood glucose	10 (29.4)	10 (33.3)	
Low HDL cholesterol	19 (55.6)	13 (43.3)	
Elevated blood pressure > 130/85 mmHg	2 (5.9)	8 (26.7)	
History of Statin Use, *n* (%)			
Before study			
Atorvastatin	22 (64.7)	23 (76.7)	
Rosuvastatin	6 (17.6)	5 (16.7)	
Simvastatin	3 (8.8)	1 (3.3)	
Statin therapy at Baseline			
Atorvastatin	34 (100.0)	30 (100.0)	
10 mg	14 (41.2)	13 (43.3)	
20 mg	14 (41.2)	8 (26.7)	
40 mg	4 (11.8)	7 (23.3)	

*Treated by medication **Metabolic syndrome definition: ≥3 of these risk factors: abdominal obesity (waist circumference > 90 cm (men) or >80 cm (women)); elevated triglycerides ≥ 1.7 mmol/L; elevated fasting blood glucose level ≥ 6.2 mmol/L; low HDL-Cholesterol level (<1.0 mmol/L (men) and <1.3 mmol/L (women)) and elevated blood pressure > 130/85 mm Hg

**Table 2:** Lipid profile.

Parameter	EZE + Statin	STAT^2 ^	*P* Value
			(Between-Group)
	*n* = 34	*n* = 30	

Baseline, mmol/L (mean ± SD)			
Total cholesterol	4.29 ± 0.62	4.57 ± 0.95	0.161
LDL-C	2.54 ± 0.53	2.77 ± 0.68	0.140
HDL-C	1.06 ± 0.27	1.10 ± 0.32	0.589
Triglycerides	1.66 ± 0.78	1.50 ± 0.66	0.386
Total cholesterol/HDL-C ratio	4.21 ± 1.00	4.67 ± 2.82	0.376

	*n* = 28	*n* = 28	

Six weeks, mmol/L (mean ± SD)			
Total cholesterol	3.75 ± 1.01	3.99 ± 0.90	0.351
LDL-C	2.08 ± 0.80	2.32 ± 0.57	0.223
HDL-C	1.07 ± 0.30	1.04 ± 0.29	0.738
Triglycerides	1.32 ± 0.53	1.44 ± 0.87	0.547
Total cholesterol/HDL-C ratio	3.64 ± 1.06	4.00 ± 1.03	0.197

	*n* = 29	*n* = 23	

Twelve weeks, mmol/L (mean ± SD)*			
Total cholesterol	3.55 ± 0.92	3.67 ± 0.50	0.563
LDL-C	1.86 ± 0.63	2.02 ± 0.39	0.294
HDL-C	1.13 ± 0.47	1.02 ± 0.28	0.351
Triglycerides	1.22 ± 0.47	1.38 ± 0.55	0.287
Total cholesterol/HDL-C ratio	3.29 ± 0.77	3.84 ± 1.15	0.046

LDL-C: low density lipoprotein cholesterol, HDL-C: high density lipoprotein cholesterol.

*12 weeks results account for 2 crossovers in treatment assignment, reflecting treatment actually received (Figure 2).

**Table 3:** Absolute and percent change in lipid parameters from baseline to 6 weeks and from baseline to 12 weeks, by treatment group.

Parameter		EZE + Statin	STAT^2^	*P* Value (Between-Group)
		*n* = 28	*n* = 28	

Six weeks, mmol/L (mean ± SD)				
Total cholesterol	Absolute change	−0.59 ± 0.82*	−0.50 ± 0.54*	0.600
	% change	−13.84 ± 17.81*	−10.93 ± 11.38*	0.469
LDL-C	Absolute change	−0.50 ± 0.74*	−0.39 ± 0.46*	0.494
	% change	−18.82 ± 26.99*	−13.55 ± 16.08*	0.378
HDL-C	Absolute change	−0.04 ± 0.12	−0.06 ± 0.15	0.602
	% change	−4.02 ± 10.63	0.42 ± 40.28	0.575
Triglycerides	Absolute change	−0.31 ± 0.75	−0.03 ± 0.66	0.152
	% change	−9.96 ± 29.24	0.73 ± 39.26	0.253
Total Chol/HDL-C	Absolute change	−0.44 ± 0.70*	−0.64 ± 2.66	0.708
	% change	−10.07 ± 15.80*	−5.34 ± 20.77	0.341

		*n* = 29	*n* = 23	

Twelve weeks, mmol/L (mean ± SD)**				
Total cholesterol	Absolute change	−0.80 ± 0.37*	−0.83 ± 0.76*	0.917
	% change	−18.06 ± 17.90*	−16.60 ± 12.62*	0.741
LDL-C	Absolute change	−0.73 ± 0.69*	−0.69 ± 0.66*	0.830
	% change	−26.62 ± 23.65*	−22.71 ± 18.19*	0.517
HDL-C	Absolute change	0.018 ± 0.29	−0.06 ± 0.19	0.257
	% change	0.48 ± 17.55	0.09 ± 38.47	0.960
Triglycerides	Absolute change	−0.38 ± 0.72*	−0.11 ± 0.54	0.141
	% change	−14.58 ± 28.78*	1.04 ± 35.20	0.084
Total Chol/HDL-C	Absolute change	−0.80 ± 0.81*	−0.95 ± 2.42	0.757
	% change	−18.05 ± 14.33*	−12.19 ± 19.69*	0.220

		*n* = 27	*n* = 23	

Twelve weeks sensitivity analysis, mmol/L (mean ± SD)***				
Total cholesterol	Absolute change	−0.77 ± 0.86*	−0.82 ± 0.76*	0.810
	% change	−17.38 ± 18.36*	−16.60 ± 12.62*	0.874
LDL-C	Absolute change	−0.72 ± 0.70*	−0.69 ± 0.66*	0.197
	% change	−26.34 ± 24.31*	−22.71 ± 18.19*	0.170
HDL-C	Absolute change	0.03 ± 0.29	−0.06 ± 0.19	0.761
	% change	1.28 ± 17.31	0.88 ± 38.47	0.864
Triglycerides	Absolute change	−0.37 ± 0.74*	−0.11 ± 0.54	0.559
	% change	−13.24 ± 29.41	1.04 ± 35.20	0.961
Total Chol/HDL-C	Absolute change	−0.80 ± 0.79*	−0.95 ± 2.42	0.125
	% change	−18.27 ± 13.77*	−12.19 ± 19.69*	0.207

LDL-C low density lipoprotein cholesterol, HDL-C high density lipoprotein cholesterol

*P value based on One-Sample *t*-test (significantly different than zero). Alpha level of 0.025 to account for multiple comparisons

**12 weeks results account for 2 crossovers in treatment assignment, reflecting treatment actually received (Figure 2)

***12 weeks sensitivity analysis excluded the 2 crossovers and was conducted only on patients maintained in their respective treatment group.

**Table 4:** Safety parameters.

Parameter		EZE + Statin	STAT^2 ^
		*n* = 34	*n* = 30
		*n* (%)^†^	*n* (%)^†^
Patients with at least one nonserious adverse event		4 (11.8)	10 (33.3)

System organ class (SOC)	Preferred term (PT)		

Eye disorder	Eye irritation	0 (0.0)	1 (3.3)
Gastrointestinal disorders	Abdominal pain	0 (0.0)	1 (3.3)
	Abdominal discomfort	0 (0.0)	3 (10.0)
General disorders and administration site conditions	Drug intolerance	1 (2.9)	1 (3.3)
Infections and infestations	Influenza	1 (2.9)	0 (0.0)
	Bronchitis	1 (2.9)	0 (0.0)
Investigations	Blood pressure increased	0 (0.0)	1 (3.3)
	Blood creatine kinase increased	0 (0.0)	3 (10.0)
Metabolism and nutrition disorders	Type 2 diabetes mellitus	1 (2.9)	0 (0.0)
Musculoskeletal and connective tissue disorders	Sensation of heaviness	0 (0.0)	1 (3.3)^ §^
	Myalgia	1 (2.9)	2 (6.7)
	Pain in extremity	0 (0.0)	1 (3.3)
Nervous system disorders	Somnolence	0 (0.0)	1 (3.3)^ §^

Patients were counted once for the corresponding preferred term and body system.

Adverse events were coded with the Medical Dictionary for Regulatory Activities version 12.0.

^†^Percentages are based on the total number of patients in each group.

^§^Adverse events occurring in (same) crossover patients.

**Table 5:** Exploratory Markers*.

Parameter		EZE + Statin	STAT^2^	*P*-Value (Between-Group)**
Baseline				

C-reactive Protein^†^ (mg/L)	Mean ± SD	2.83 ± 2.95	3.63 ± 5.35	0.567
	Median (Min; Max)	1.50 (0.00; 13.00)	2.00 (0; 27.00)	
Interleukin-6^†^ (pg/mL)	Mean ± SD	3.63 ± 3.55	3.25 ± 1.92	0.883
	Median (Min; Max)	3.05 (1.00; 20.00)	2.92 (0.00; 9.00)	
Apolipoprotein-B (g/L)	Mean ± SD	0.89 ± 0.18	0.93 ± 0.20	0.543
	Median (Min; Max)	0.90 (0.57; 1.30)	0. 91 (0.61; 1.34)	
Apolipoprotein-A1 (g/L)	Mean ± SD	1.42 ± 0.20	1.44 ± 0.29	0.780
	Median (Min; Max)	1.43 (1.08; 1.89)	1.39 (0.85; 2.10)	
ApoB/ApoA1	Mean ± SD	0.64 ± 0.16	0.66 ± 0.15	0.700
	Median (Min; Max)	0.62 (0.33; 1.07)	0.62 (0.41; 1.00)	
Adiponectin^†^ (mg/L)	Mean ± SD	8.53 ± 4.68	8.92 ± 3.33	0.276
	Median (Min; Max)	6.96 (3.89; 24.90)	7.82 (4.20; 16.59)	
Leptin^†^ (ng/mL)	Mean ± SD	26.96 ± 30.16	18.48 ± 13.14	0.623
	Median (Min; Max)	17. 90 (2.00; 1.42)	13.97 (3.00; 46.00)	

12 weeks mmol/L				

C-reactive Protein^†^ (mg/L)	Mean ± SD	2.26 ± 3.24	3.72 ± 4.31	0.079
	Median (Min; Max)	1.10 (0.00; 16.00)	1.90 (0.00; 17.00)	
Interleukin-6^†^ (pg/mL)	Mean ± SD	2.89 ± 1.14	4.90 ± 5.67	0.306
	Median (Min; Max)	3.08 (1.00; 5.00)	3.11 (1.00; 24.00)	
Apolipoprotein-B^†^ (g/L)	Mean ± SD	0.74 ± 0.22	0.78 ± 0.12	0.110
	Median (Min; Max)	0.67 (0.44; 1.41)	0.79 (0.55; 1.06)	
Apolipoprotein-A1 (g/L)	Mean ± SD	1.39 ± 0.15	1.41 ± 0.28	0.754
	Median (Min; Max)	1.40 (1.07; 1.66)	1.42 (0.82; 1.98)	
ApoB/ApoA1^†^	Mean ± SD	0.54 ± 0.17	0.58 ± 0.16	0.252
	Median (Min; Max)	0.50 (0.30; 1.04)	0.55 (0.38; 1.05)	
Adiponectin (mg/L)	Mean ± SD	7.72 ± 3.06	8.14 ± 2.98	0.638
	Median (Min; Max)	7.35 (3.48; 17.42)	7.36 (3.99; 14.76)	
Leptin^†^ (ng/mL)	Mean ± SD	27.51 ± 41.14	23.41 ± 18.02	0.832
	Median (Min; Max)	19.10 (3.00; 206.00)	21.40 (2.00; 65.00)	

*Analysis performed on those patients with available data

**Mann-Whitney *U* non-parametric test for independent samples or independent samples *t*-test, depending on distribution assessed with Shapiro-Wilk test of normality.

^†^For parameters not normally distributed, the median rather than the mean should be preferred.
